# Patients with renal transplant and moderate-to-severe LUTS benefit from urodynamic evaluation and early transurethral resection of the prostate

**DOI:** 10.1007/s00345-021-03799-y

**Published:** 2021-09-04

**Authors:** Marialaura Righetto, Mariangela Mancini, Daniele Modonutti, Arturo Calpista, Paolo Beltrami, Fabrizio Dal Moro

**Affiliations:** 1 Department of Surgical, Oncological and Gastroenterological Sciences, University of Padova, Padova, Italy; 2 Urological Clinic, University Hospital of Padova, Padova, Italy

**Keywords:** Lower urinary tract symptoms, Renal transplantation, Bladder outlet obstruction, Urodynamic study, Graft failure, Voiding functional outcomes

## Abstract

**Purpose:**

To assess long-term renal function and micturition pattern of males submitted to transurethral resection of the prostate (TURP) for moderate-to-severe lower urinary tract symptoms (LUTS) after renal transplantation (RT). To investigate the role of clinical and urodynamic (UD) parameters for bladder outlet obstruction (BOO) diagnosis in these patients.

**Methods:**

Retrospective data analysis of ≥ 50 years old patients who underwent RT between 01/2005 and 12/2016. Patients with moderate-to-severe LUTS after RT who underwent a urologic evaluation and a UD study were included. TURP was performed in case of BOO diagnosis. Kidney function and micturition patterns were evaluated before, 3, 12, 24, 36, and 48 months after TURP. Predictors of BOO were assessed at univariable and multivariable logistic regression models. Statistical analysis was performed with STATA16.

**Results:**

233 male patients ≥ 50 years underwent RT. 71/233 (30%) patients developed voiding LUTS. 52/71 (73%) patients with moderate-to-severe LUTS underwent UD. TURP was performed in 36/52 (69%) patients, with BOO diagnosis. Median (interquartile range) follow-up was 108 (75–136) months. Maximum flow at flowmetry (Qmax), International Prostate Symptom Score and post-voided residual volume improved significantly after surgery. Serum creatinine decreased and glomerular filtration rate improved significantly at follow-up, especially when TURP was performed ≤ 6 months from RT. At the multivariable model, bladder capacity ≥ 300 mL (OR = 1.74, CI 95% 1.03–3.15, *p* = 0.043) and detrusor pressure at Qmax (OR = 2.05, CI 95% 1.48–3.02, *p* = 0.035) were the independent predictors of BOO.

**Conclusion:**

RT patients with moderate-to-severe LUTS at risk for BOO and graft failure are better identified by UD than clinical parameters. Bladder capacity and voiding pressure are key for the early diagnosis of BOO.

## Introduction

Advances in life expectancy have led to an increase in the number of older people with end-stage renal disease (ESRD) undergoing renal transplantation (RT). In the United States, in 2016, 21.3% of transplant recipients were 65–74 years old [1]. In Europe, more than 8% of RT is performed in recipients aged > 75 years [2]. Age-related issues become critical for RT success. Lower urinary tract symptoms (LUTS) due to bladder outlet obstruction (BOO) in males linearly increase with age. The incidence of moderate-to-severe voiding LUTS, classified according to the International Prostate Symptoms Score (IPSS) score [3], is about 30–40% in men aged > 50 years [4, 5]. This problem is underdiagnosed among RT recipients, because of oligoanuresis. LUTS can arise after diuresis restoration, with a risk to graft function. Urodynamic evaluation (UD) is required for BOO diagnosis [6]. However, UD is not routinely recommended in the preoperative workup of RT. Transurethral resection of the prostate (TURP) is the standard treatment for BOO [7] and could improve the long-term graft function in RT patients with BOO. Few studies have been published on the role of TURP in transplanted patients, with none on the relevance of combining clinical and UD parameters for a correct early diagnosis. We investigated these points in our study.

## Materials and methods

### Population and data source

We retrospectively analyzed data from male patients with ESRD who received an RT between 01/2005 and 12/2016 at the University Hospital of Padua, Italy. Data were collected from hospital records, outpatient visits, and hospital admissions after TURP. Before RT, patients were screened with prostate volume at trans-rectal ultrasonography, total prostate-specific antigen (PSA), and cystography (to exclude the presence of urethral stenosis). After RT, patients ≥ 50 years with voiding LUTS who underwent urologic evaluation were considered for the study. Patients with Eastern Cooperative Oncology Group performance status (ECOG PS) ≥ 3, New York Heart Association (NYHA) class [8] ≥ 3, clinical or UD diagnosis of storage LUTS only, and prostate cancer patients were excluded, as well as patients with LUTS secondary to neurological disorders. Patients with moderate-to-severe LUTS, according to the IPSS score underwent UD. Antimicrobial prophylaxis was performed with oral fosfomycin trometamol (3 g) the night before and after UD.

**Fig. 1:**
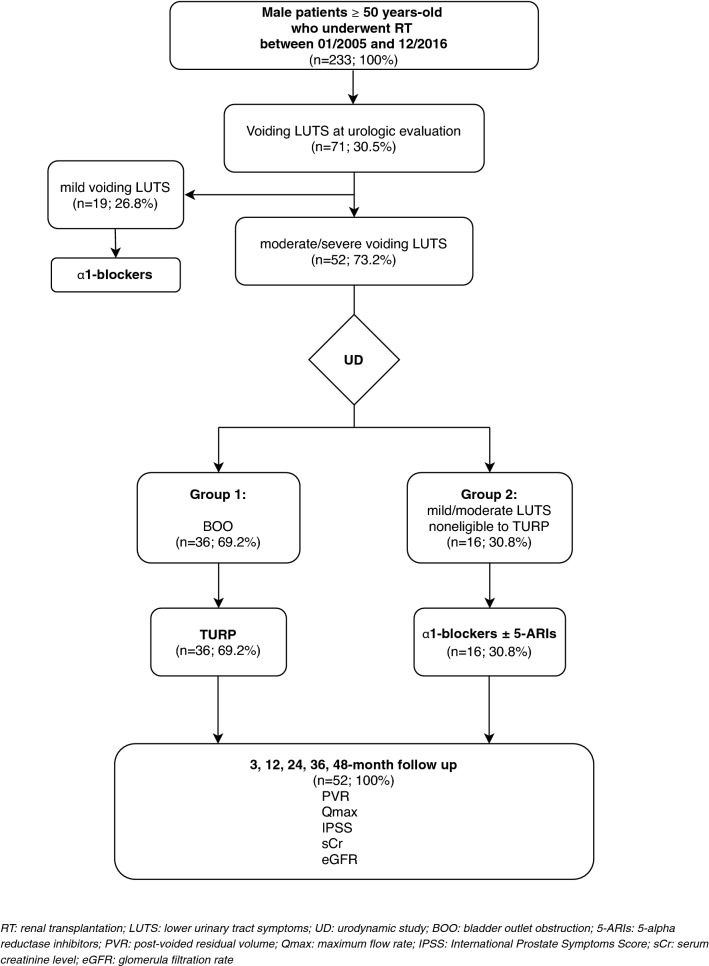
Flow-chart of the population considered for the study design

Two groups of patients were identified at UD. Group 1 included patients with moderate-to-severe LUTS and BOO diagnosis, patients with a post-voided residual volume (PVR) > 1/3 of their bladder capacity, and patients with acute urinary retention requiring a permanent bladder catheter. All these patients were submitted to TURP. Group 2 included patients with mild-moderate LUTS without BOO at UD, who received α1-blockers $\pm$ 5α-reductase inhibitors. We treated separately patients without BOO at UD with voiding symptoms due to urethral stenosis or detrusor hypocontractility. TURP was performed under spinal or general anesthesia, with a bipolar 27 F resectoscope. Patients in both groups were evaluated at 3, 12, 24, 36, and 48 months with history, IPSS, renal function, uroflowmetry (UFM), and PVR. (Fig. 1).

### Endpoints and outcomes

Our primary endpoint was to evaluate renal function and graft survival at the follow-up visits after TURP. Graft survival was defined as the time from RT to the return to dialysis. Renal function was evaluated by serum creatinine (sCr) and estimated glomerular filtration rate (eGFR) values. We then assessed the urinary functional improvement after TURP, defined as a PVR ≤ 50 mL with a ≥ 3-point improvement of the IPSS or a maximum flow rate (Qmax) ≥ 15 mL/min at UFM. Finally, we investigated the impact of the time-to-TURP on renal function.

### Covariates

Demographic, clinical, and laboratory variables were collected including age, BMI (18.5–24.9; 25–29.9;  ≥ 30 kg/m^2^), ECOG PS, NYHA class, 24-h urinary output (anuria  ≤ 100 mL/day; oliguria 101–400 mL/day; preserved diuresis  > 400 mL/day [9]), type of dialysis, stay-on-dialysis (months), prostate volume at trans-rectal ultrasonography, total PSA, ultrasound PVR, sCr and eGFR (derived using Chronic Kidney Disease Epidemiology—CKD-EPI [10]). For patients with an indwelling bladder catheter, we considered the sCr and eGFR levels before the catheterization. LUTS were categorized into mild (IPSS score 1–7), moderate (IPSS score 8–19), and severe (IPSS score 20–35). We considered the following UD variables: bladder capacity (< 100, 100–300,  > 300 mL), Qmax (mL/s), detrusor pressure at Qmax (PdetQmax; cmH2O), PVR (< 1/3 of bladder capacity,  > 1/3 of bladder capacity). BOO was defined according to the Abrams-Griffiths nomogram [11].

### Statistical analysis

Continuous variables were reported using mean (standard deviation; SD) or median (interquartile range; IQR) if they had a normal or non-normal distribution at the Shapiro–Wilk test, respectively. Categorical variables were reported using frequencies and percentages. The differences between baseline characteristics of continuous variables in Group 1 and 2 were examined with the 2-sample *t* test for means and with the Wilcoxon test for medians. Differences between baseline characteristics of categorical variables in the two groups were evaluated with Fisher’s exact test. We used the χ^2^ test to compare the IPSS score and PVR before and after TURP. The paired *t *test was used to compare sCr levels, Qmax, and eGFR values before and after TURP. The Friedman’s test was used to compare sCr, Qmax, and eGFR before and after 3, 12, 24, 36, and 48 months. We performed a univariable logistic regression to test the prognostic significance of the covariates as predictors of BOO. Finally, variables with a significance level of ≤ 0.15 in the univariable analysis were included in the multivariable model. Statistical analysis was performed with STATA^®^ software (StataCorp, College Station, TX, USA-version 16). Comparisons were carried out at the 95% confidence level (CI95%); a two-sided *p *value < 0.05 was considered statistically significant.

## Results

RT was performed in 233 male patients ≥ 50 years old. 71 (30.5%) underwent urologic evaluation after RT. 52/71 (73%) were sent to UD, the other 19/71 (27%) patients presented mild LUTS treated with medical therapy. 36/52 (69%) patients had a BOO diagnosis at UD and underwent TURP. The median (IQR) follow-up was 108 (75–136) months.

Baseline characteristics of patients who underwent UD are shown in Table 1.

**Table 1:** Baseline characteristics of the RT recipients who underwent UD (*n* = 52)

**Baseline characteristics**	**Group 1**	**Group 2**	***p*** **value**	**UD characteristics**	**Group 1**	**Group 2**	***p*** **value**
	***n*** **= 36**	***n*** **= 16**			***n*** **= 36**	***n*** **= 16**	
Age (years) (mean [SD])	64 (13.8)	58 (7.2)	*0.043*	Bladder capacity (*n* [%])			*0.65*
				< 100 mL	0 (0)	0 (0)	
				100–300 mL	13 (36.1)	7 (43.8)	
				> 300 mL	23 (63.9)	9 (56.2)	
BMI (*n* [%])			*0.88*	Bladder sensitivity (*n* [%])			
18.5–24.9 kg/m2	1 (2.8)	1 (6.2)		Normal	15 (41.7)	6 (37.5)	*0.73*
25–29.9 kg/m2	34 (94.4)	15 (93.8)		Augmented	14 (38.9)	9 (56.3)	
≥ 30 kg/m2	1 (2.8)	0 (0)		Reduced	7 (19.4)	1 (6.2)	
Comorbidities (*n* [%])				Bladder compliance (*n* [%])			
NYHA 1	16 (44.4)	13 (81.3)	*0.85*	Normal	22 (61.2)	8 (50)	*0.62*
2	20 (55.6)	3 (18.7)		Augmented	7 (19.4)	2 (12.5)	
Diabetes				Reduced	7 (19.4)	6 (37.5)	
Yes	15 (41.7)	9 (56.2)	*0.92*				
No	21 (58.3)	7 (43.8)					
ECOG PS 0	32 (88.9)	16 (100)	*0.84*				
1	3 (8.3)	0 (0)					
2	1 (2.8)	0 (0)					
Dialysis (*n* [%])							< *0.001*
Hemodialysis	26 (72.2)	13 (81.3)	*0.56*	Detrusor contraction (*n* [%])	14 (38.9)	0 (0)	
Peritoneal dialysis	10 (27.8)	3 (18.7)		Hyperactivity	0 (0)	0 (0)	
Diuresis (*n* [%])				Underactivity			
Preserved	12 (33.3)	3 (18.7)	*0.22*				
Oliguric	6 (16.7)	0 (0)					
Anuric	18 (50)	13 (81.3)					
Stay-on-dialysis (months) (median [IQR])	37 (1–76)	22 (1–30)	*0.045*	Urinary incontinence (*n* [%])			< *0.001*
				Stress incontinence	3 (8.3)	0 (0)	
				Urge incontinence	0 (0)	0 (0)	
sCr (umol/L) (mean [SD])	221 (92)	226 (35)	*0.95*	Voiding phase (mean [SD])			< *0.001*
				Qmax (mL)	8.9 (5.8)	18.9 (4.6)	
				PdetQmax (mmHg)	0 (0)		
eGFR (mL/min/1.73 m^2^) (mean [SD])	40 (22)	38 (22)	*0.87*	PVR (n [%])	57.5 (31)	32.2 (6.1)	*0.12*
				< 1/3 bladder capacity	19 (52.8)	16 (100)	
				> 1/3 bladder capacity	17 (47.2)	0 (0)	
Prostatic volume at TRUS (cm3) (median [IQR])	40 (30–45)	25 (18–40)	*0.033*	BOO (n [%])			*0.041*
				Unobstructed	0 (0)	16 (100)	
				Obstructed	27 (75)	0 (0)	
				Equivocal	9 (25)	0 (0)	
Total PSA (ng/mL) (mean [SD])	1.7 (1.4)	1.1 (0.7)	*0.088*				
Qmax at flowmetry (mL) (mean [SD])	9.2 (6.7)	10.8 (6.1)	*0.86*				
Ultrasound PVR (mL) (median [IQR])	130 (90–145)	75 (50–135)	*0.042*				
IPSS score (*n* [%])			*0.035*				
IPSS score 1–7	0 (0)	14 (87.5)					
IPSS score 8–19	21 (58.3)	2 (12.5)					
IPSS score 20–35	15 (41.7)	0 (0)					

*UD* urodynamic study, *RT* renal transplantation, *LUTS* lower urinary tract symptoms, *TURP* transurethral resection of the prostate, *IQR* interquartile-range; SD: standard deviation, *BMI* body mass index, *NYHA* New York Heart Association functional classification, *ECOG PS* Eastern Cooperative Oncology Group Performance Status, *TRUS* trans-rectal ultrasonography, *PVR* post-voided residual volume, *IPSS* International Prostate Symptoms Score, *sCr* serum creatinine, *eGFR* estimated glomerular filtration rate, *Qmax* maximun flow rate, *PdetQmax* detrusor pressure at Qmax, *BOO* bladder-outlet obstruction

None of the patients developed symptomatic urinary tract infection (UTI) or fever after UD.

As compared to Group 2, patients in Group 1 were older, with a longer median stay-on-dialysis, a higher median prostatic volume and PVR, a lower mean Qmax at UD, a higher mean PdetQmax, and median PVR. 75% of them had BOO at the Abrams-Griffiths nomogram, 25% had equivocal results. The median time-to-TURP was 9 (IQR 2–12) months. Group 2 patients benefited at follow-up to drug therapy alone and did not undergo TURP.

No patient in Group 1 underwent a re-TURP during follow-up. Functional and renal outcomes of Group 1 patients are shown in Table 2. Three months after TURP, the median (IQR) IPSS and the PVR significantly decreased to 3 (1–6) and 0 (0–0) mL, respectively (*p* < 0.001), while Qmax was significantly higher (*p* < 0.001). 53 and 47% of the patients doubled their Qmax at 3 and 12 months, respectively, and 25% tripled it at 12 months. At the 3-month visit, 97% of the patients had a PVR ≤ 50 mL and a ≥ 3-point IPSS improvement, while 89% of the patients had a PVR ≤ 50 mL and a Qmax ≥ 15 mL/min. sCr levels were significantly lower than pre-TURP up to 36 months (*p* < 0.001), while the eGFR improved significantly only at the 24-month follow-up visit (*p* = 0.047). Moreover, at the first follow-up visit, despite similar baseline values, sCr levels were significantly lower and eGFR was significantly higher in Group 1 patients than in Group 2 (sCr 171 ± 93 vs. 255 ± 89 mmol/L, *p* < 0.001; eGFR 45 ± 18 vs. 33 ± 11 mL/min/1.73 m^2^, *p* = 0.045). These differences did not remain significant at 48 months for sCr (199 ± 136 vs. 175 ± 102 mmol/L; *p* = 0.43), and at 36 months for eGFR (46 ± 18 vs. 44 ± 13 mL/min/1.73 m^2^; *p* = 0.76).

**Table 2:** Functional and renal outcomes of Group 1 patients (*n* = 36)

	Pre-TURP	3 months	12 months	24 months	36 months	48 months
IPSS	16 (10–20; 9–23)^a^	3 (1–6; 0–6)	4 (0–6; 0–6)	2 (0–5; 0–7)	3 (0–4; 0–6)	3 (0–4; 0–5)
Median (IQR min–max)		*p* < *0.001*	*p* < *0.001*	*p* < *0.001*	*p* < *0.001*	*p* < *0.001*
Qmax (mL/s)	9.6 (5.5)a	19.2 (3.2)	19.9 (2.7)	19.8 (2.7)	19.5 (3.5)	19.1 (3.3)
Mean (SD)		*p* < *0.001*	*p* < *0.001*	*p* < *0.001*	*p* < *0.001*	*p* < *0.001*
PVR (mL)	130 (90–145; 70–400)a	0 (0–0; 0–75)	0 (0–30; 0–50)	0 (0–0; 0–50)	0 (0–0; 0–50)	0 (0–0; 0–50)
Median (IQR min–max)		*p* < *0.001*	*p* < *0.001*	*p* < *0.001*	*p* < *0.001*	*p* < *0.001*
sCr (μmol/L)	258 (169)a	171 (93)	154 (56)	158 (61)	157 (55)	199 (136)
Mean (SD)		*p* < *0.05*	*p* < *0.05*	*p* < *0.001*	*p* < *0.001*	*p* = *0.88*
eGFR (mL/min/1.73 m^2^)	40 (22)a	45 (18)	47 (20)	48 (20)	46 (18)	42 (21)
Mean (SD)		*p* = *0.12*	*p* = *0.09*	*p* = *0.047*	*p* = *0.09*	*p* = *0.97*

*RT* renal transplantation, *TURP* transurethral resection of the prostate, *IQR* interquartile-range, *SD* standard deviation; IPSS: International Prostate Symptoms Score, *Qmax* maximum flow rate, *PVR* post-voided residual volume, *sCr* serum creatinine; eGFR: estimated glomerular filtration rate

^a^Variable used as reference

16/36 (44%) patients underwent TURP within 6 months from the RT (9 of these within 1 month). Unlike patients who underwent TURP later, all these patients had a PVR > 1/3 of their bladder capacity, and a tendency towards a longer median (IQR) stay-on-dialysis (49 [18–76] vs. 35 [1–37] months; *p* = 0.057) and a higher median (IQR) prostatic volume (37 [26–45] vs. 24 [15–31] cm^3^, *p* = 0.053). At 3 months, the IPSS score was similar in the two groups. However, there was a significant improvement in the mean (SD) sCr and eGFR levels only in patients who underwent TURP earlier (sCr 154 ± 48 vs. 186 ± 91 mmol/L, *p* = 0.046; eGFR 47 ± 6 vs. 38 ± 4 mL/min/1.73 m^2^, *p* = 0.041). Interestingly, patients who underwent TURP within 1 month after RT, had a higher 3-months eGFR level (42 ± 2 mL/min/1.73 m^2^), albeit this trend did not reach significance, probably for the low sample size.

None of the 52 patients with moderate-to-severe LUTS underwent re-transplantation. 4/52 (8%) patients returned to hemodialysis. The graft survival after TURP was 100% at 12 months and 97% at 24 months.

Long stay-on-dialysis, PSA value, Qmax at UFM, high bladder capacity and PdetQmax predicted BOO at the univariable analysis. At the multivariable model, high bladder capacity (OR = 1.74, CI 95% 1.03–3.15, *p* = 0.043) and PdetQmax (OR = 2.05, CI 95% 1.48–3.02, *p* = 0.035) were the two independent predictors of BOO, while the Qmax value was not (Table 3). PSA value increased BOO risk by three folds, but this data did not reach significance, probably for the low sample size.

**Table 3:** Univariable and multivariable logistic regression model of BOO in RT recipients with moderate-to-severe LUTS (*n* = 52)

Univariable analysis	OR	95% CI	*p *value
Age (years)	1.29	0.13–2.52	0.81
NYHA			
1^a^			
2	1.33	0.77–1.61	0.51
Diuresis	0.57	0.48–1.21	0.53
Preserved^a^ oliguric/anuric	2.18	0.48–2.36	0.42
Indwelling bladder catheter	1.18	0.33–1.48	0.77
Stay-on-dialysis (months)			
≤ 36^a^			
> 36	1.33	1.17–1.78	0.038
PSA (ng/mL)	2.13	1.33–5.24	0.022
Qmax at flowmetry (mL/s)	1.18	1.03–2.29	0.036
Prostatic volume (cm^3^)	0.96	0.88–1.06	0.44
Bladder capacity at UD (mL)			
≤ 300^a^			
> 300	1.54	1.02–2.25	0.033
Sensibility at UD	0.73	0.58–2.91	0.78
Qmax at UD (mL/s)	1.37	0.16–1.43	0.57
PdetQmax (cmH_2_O)	1.21	1.06–2.36	0.041
PVR			
< 1/3 bladder capacity^a^			
> 1/3 bladder capacity	1.45	0.79–1.68	0.69

Multivariable analysis	OR	95% CI	*p *value
Bladder capacity at UD (mL)			
≤ 300^a^			
> 300	1.74	1.03–3.15	0.043
PSA (ng/mL)	3.04	0.68–13.7	0.16
Qmax at flowmetry (mL/s)	2.51	0.33–4.69	0.76
PdetQmax (cmH_2_O)	2.05	1.48–3.02	0.035

*BOO* bladder-outlet obstruction, *RT* renal transplantation, *LUTS* lower urinary tract symptoms, *NYHA* New York Heart Association functional classification, *Qmax* maximun flow rate, *UD* urodynamic study, *PdetQmax* detrusor pressure at Qmax, *PVR* post-void residual volume

^a^Variable used as reference

## Discussion

ESRD incidence increases with age [12]. Improvements in surgical techniques and immunosuppressive therapies have increased the success rate of RT in older patients, who frequently have undiagnosed LUTS and could present BOO once normal micturition is restored. LUTS onset in these patients leads to high-pressure bladder storage, increased PVR, dysfunctional detrusor contraction, hydronephrosis, and graft failure [13].

A retrospective cohort study on 23.622 recipients [14] reported an incidence of 9.7% and 7.3% of BPH and TURP, respectively, 3 years after RT, without stratifying by age. More recently, a retrospective study on 131 recipients, showed a benign prostatic obstruction prevalence of 58% between 60 and 70 years, and 71% above 70 years [15].

Reported series of TURP for BOO in RT recipients are scarce, with a short median follow-up, difficult to compare in terms of patients’ characteristics, BOO definitions, and outcomes [13, 16–18]. Particularly, there is a lack of standardization in the BOO definition, as UD is not considered as a key test for patients presenting LUTS after RT.

We included in our study male patients aged ≥ 50 years at the time of RT, who showed, similarly to other studies [16, 17, 19], a 30% incidence of LUTS after RT. Moreover, 51% of RT recipients with LUTS underwent TURP. All recipients with moderate-to-severe LUTS were submitted to UD assessment, and only in the case of BOO diagnosis, a TURP was performed. In our study, UD was instrumental in sorting outpatients with BOO who required TURP, a result we could not find in previously published studies. 31% of patients who underwent UD did not show BOO and received only oral drugs. Notably, they never underwent TURP at subsequent follow-up. Moreover, UD turned out to be a safe exam, with no patient having a postprocedural UTI. This data is in accordance with recent evidence, which no longer proposes antibiotic prophylaxis even in high-risk immunosuppressed patients [20].

Performing TURP in RT recipients was clinically beneficial, in our study, according to the long-term renal and functional outcomes. Firstly, we found an improvement in sCr and eGFR levels up to 36 and 24 months from TURP, respectively. At subsequent follow-ups, sCr and eGFR levels started to get worse, probably as the result of the natural history of transplanted kidneys. Renal function improvement has been reported in previously published series [13, 16, 17]. Our study confirms this data with a higher median follow-up and highlights the fact that the improvement is higher for patients submitted to TURP than for those treated with drug therapy. An additional detail, at the 3-month visit the improvement in sCr and eGFR levels was higher in patients submitted to TURP soon after RT (≤ 6 month), as compared to patients who underwent TURP later. Our data suggest that, in agreement with what was previously reported [17, 21], there is no reason to delay TURP in patients with moderate-to-severe LUTS and BOO after RT, and that earlier TURP (and ‘very-early’ ≤ 1-month TURP) effectively improves graft function. Finally, functional outcome improved after TURP. Three months after, indeed, more than 97% of patients had a significant decrease in their IPSS score and in PVR, with an increase in Qmax, a trend stable at subsequent follow-ups.

We performed a logistic regression analysis to search for predictors of BOO specific for transplanted patients. Two clinical parameters (PSA value and Qmax at flowmetry) correlated with BOO at univariable analysis. However, at multivariable analysis, they were not independent predictors of BOO. Moreover, differently from what had previously been reported by Gratzke et al. [22], patients’ age and stay-on-dialysis were not predictors of BOO or TURP after RT. Therefore, no preoperative clinical parameter, evaluated with non-invasive tests, was able to accurately predict the presence of BOO. On the other hand, we did find that the onset of BOO was significantly correlated only to bladder capacity and PdetQmax, both measured at UD. Particularly, patients with a higher bladder capacity had a 70% higher risk of being ‘TURPed’ than those with a lower one. We could not correlate these observations with any other reported series. According to these results, UD could be offered instead of other clinical tests, as a better tool to reliably identify BOO in RT patients with moderate-to-severe LUTS.

Our study is limited by its retrospective nature, the lack of significance in some statistics can be due to the limited numbers of cases.

## Conclusions

RT patients with moderate-to-severe LUTS at high-risk for BOO and, potentially, graft failure, are better identified by UD than clinical parameters. Patients without BOO at the Abrams-Griffiths nomogram can be safely treated with drug therapy alone. Urodynamic evaluation of bladder capacity and bladder voiding pressure is critical for BOO reliable diagnosis in these patients. Furthermore, TURP performed early after RT could better improve renal function.

## Data Availability

The datasets generated during and/or analyzed during the current study are available from the corresponding author upon reasonable request.
